# Resolving biofilm topography by native scanning electron microscopy

**DOI:** 10.14440/jbm.2017.173

**Published:** 2017-04-25

**Authors:** Neta Raab, Ido Bachelet

**Affiliations:** 1 Augmanity, Rehovot, Israel; 2 Faculty of Life Sciences, Bar-Ilan University, Ramat-Gan, Israel

**Keywords:** biofilm, scanning electron microscopy, *Bacillus subtilis*

## Abstract

Scanning electron microscopy (SEM) is a powerful tool for structural analysis, but it requires biological samples to undergo lengthy, chemically-complex multi-step preparation procedures, arguably altering some features in the sample. Here we report an ultra-rapid and chemical-free technique for visualizing bacterial biofilms at their native state. Our technique minimizes the time interval from culture to imaging to approximately 20 min, while producing high-resolution images that enable the detection of a variety of topographic features such as bacterial chains, and resolving cells from matrix. We analyzed images obtained from *Bacillus subtilis* biofilms, demonstrate the usefulness of this technique for multiple types of image analysis, and discuss its potential to be improved and adapted to other types of biological samples.

## INTRODUCTION

Scanning electron microscopy (SEM) is a powerful tool for structural analysis in a variety of fields such as physics, electronics, and chemistry. SEM is also widespread in biology [1,2], however its use to study live tissues and cells is hindered by the biologically-incompatible requirements that sample visualization is done in vacuum, and for the sample to be conducting. To undergo SEM, biological samples must undergo a sequence of procedures that is time-consuming, labor-intensive, and chemically hazardous. Standard SEM protocols make routine use of highly toxic and corrosive materials such as osmium tetroxide [3], cacodylic acid, tannic acid, guanidine hydrochloride, *etc*. which pose serious health hazards [4]. Finally, the samples are sputtered with conducting materials such as gold or iridium. The procedures take many hours during which samples are passed through multiple substances with varying water content, inevitably undergoing changes in the process. It is plausible that certain structural features are modified or damaged during this process, or sputtering could conceal some features due to inadvertently excessive coating, and that such modifications are easily overlooked in the absence of additional reference methods for sample visualization in SEM.

Bacterial biofilms are of growing interest for their medical and environmental impact [5]. We are interested in studying bacterial biofilms for bioengineering applications that require integration of biofilms into other materials and matrices, with structural analysis in SEM being a crucial part of these studies. We found the current protocols for biological sample preparation for SEM inadequate for most of our settings, for example due to chemical incompatibility of the protocol to the biofilm-material hybrid. We therefore experimented to develop alternative methods.

Our requirements from these methods were as follows: (1) they must be rapid and include a minimum number of steps to avoid prolonged sample manipulation; (2) they must be as chemically pristine as possible, to avoid sample modification but also to improve human safety; and (3) they must provide sufficiently informative resolution, in terms of biofilm structure. A resolution trade-off seems inescapable here; however, we wished to examine exactly how much resolution needs to be sacrificed in order to visualize the sample as natively as possible.

Here we present a method for ultra-rapid, native SEM for bacterial biofilms used topographic analysis of *Bacillus subtilis* (*B. subtilis*) biofilms. Below certain adjustments and potential improvements are discussed.

## MATERIALS AND METHODS

### Bacterial strains

*B. subtilis* (strain NCIB3610) were a kind gift from I. Kolodkin-Gal (Weizmann Institute of Science, Rehovot, Israel) and cultured in defined medium (MSgg) at 23°C for 72 h.

### Sample preparation

Support discs were cut from very thin metal nets to sizes that fit culture wells, and were sterilized in 70% ethanol followed by drying over open fire before placed in wells. Cultivations of the biofilms were performed in 12 and 24 well plates containing the supports. Bacterial biofilms were gently removed using the supports and placed directly on SEM stubs. Samples were transferred to a glass desiccator containing silica gel and vacuumed at low pressure with a KNF N86KN.18 diaphragm vacuum pump for 10–20 min. At this point they were taken into the SEM for imaging. Alternatively, biofilms were first fixed in the wells by placing a filter paper soaked in glutaraldehyde (grade I, 8% v/v in water) between the well and lid and incubated for 60 min at room temperature, and then removed, attached to stubs, and dried as mentioned above. For frozen samples, biofilms with or without fixation were removed, placed on stubs, and instead of drying directly placed in a container with dry ice for 5 min, followed immediately by transferring to the sample holder that has been previously cooled to **−**25°C).

### SEM

Samples were visualized on a PhenomWorld ProX scanning electron microscope with optical magnification range of 20–135 ×, electron magnification range of 80–130000 ×, maximal digital zoom of 12 ×, acceleration voltages of 5, 10, and 15 kV, backscattered electron detector (BSD) and energy dispersive X-ray spectrometer (EDS) detectors, with nominal resolution of 10 nm or less. The microscope has a temperature controlled sample holder (temperature range **−**25°C to 50°C).

### Image analysis

Images were analyzed by FIJI using the following features: plot profile, 3D surface, directionality analysis (local gradients) with enhance local contrast.

## RESULTS

**Figure 1.**
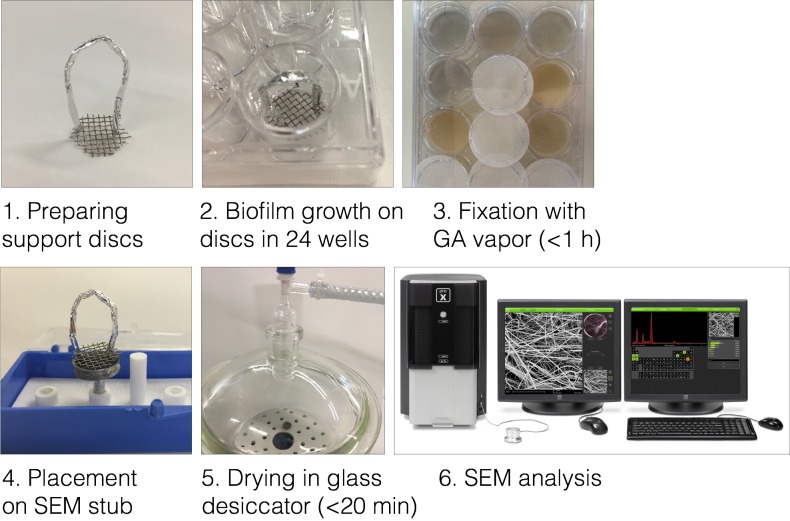
Visual representation of the procedure. Support discs are prepared from commercially available metal nets, with handles fashioned from aluminum wire or foil. The discs are placed in plate wells and sterilized. Fixation is done by placing glutaraldehyde (GA)-soaked filter paper between the plate lid and wells. Support disc with biofilm is placed on SEM stub with handles removed, and placed inside desiccator for drying. Sample is then ready for SEM acquisition and analysis.

The main goal in this study was to maintain the sample as pristine as possible to allow for the creation of authentic images. Several aspects of the method we propose here were designed with this goal in mind. First, the support discs enable lifting of the biofilms with almost no perturbation as can be verified visually, as compared to lifting it with tweezers which inevitably alters the native structure. Second, sample is only dried using a low-power pump to keep it just below the dew point where water vapor pressure is gradually decreased due to absorption by the hygroscopic silica gel. This prevents harsh sample desiccation and breaks or deformations caused by it, as is the case when the biofilm is exposed to various solvents and materials. Above all, an effort was made in designing this method to shorten as much as possible the time interval from culture to microscope. A complete visual representation of this procedure is described in **Figure 1**.

**Figure 2.**
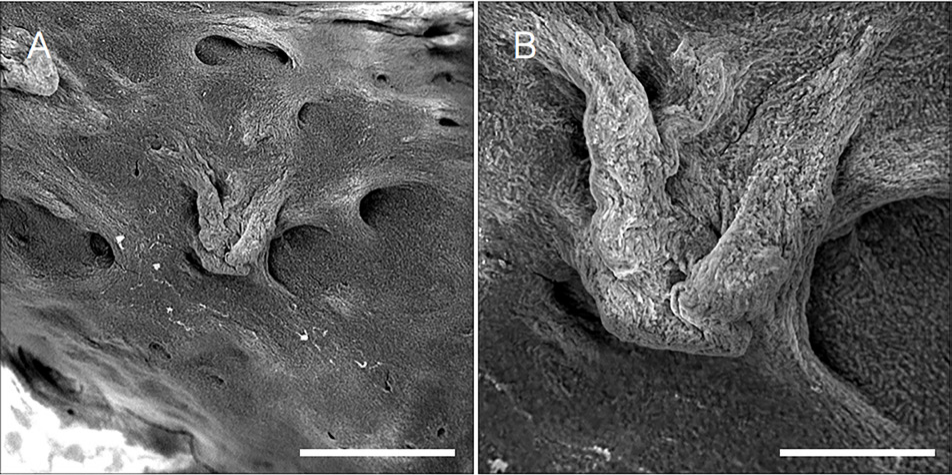
Large and small scale topography of *B.* *subtilis* biofilm visualized by native SEM. **A.** Large scale topography of ***B.*** *subtilis* (Scale bar = 100 µm). **B.** Small scale topography of ***B.*** *subtilis* (Scale bar = 30 µm). Samples were lifted from medium on support discs, placed onto SEM stubs, dried for up to 20 min, and visualized by SEM.

**Figure 3.**
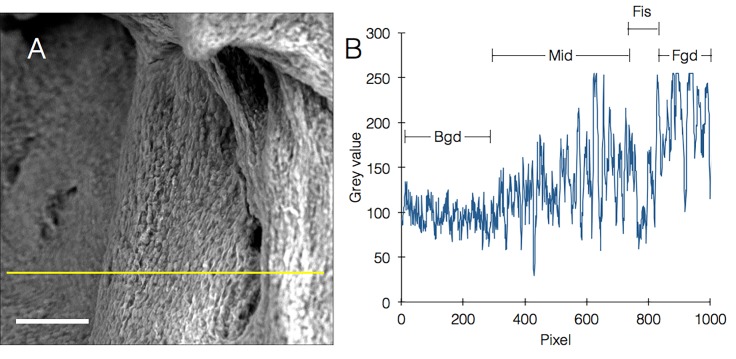
Visualizing and measuring biofilm topography in native SEM. **A.** Biofilm image acquired by native SEM. Yellow line was drawn for profile plot (B) (scale bar = 10 µm). **B.** Profile plot from (A), showing all features of the image: Bgd, background; Mid, middle segment, note slope of the mean height of this segment; Fis, fissure; Fgd, foreground segment.

*Bacillus* biofilms exhibit rich topography driven by many factors including shape of the environment [6] and mechanical forces created within the biofilm [7]. These features can be clearly visualized using our technique (**Fig. 2** and **Fig. S1**). Moreover we were able to measure and quantify height levels across the image including slopes and fissures (**Fig. 3A** and **3B** and **Fig. S2**). Our technique does not make use of sputtering so the sample becomes slightly charged after a certain imaging duration, which increases the ability to discern heights since higher surfaces are charged first. In case charging is undesired, a discussion of relevant adjustments that we propose can be found below.

**Figure 4.**
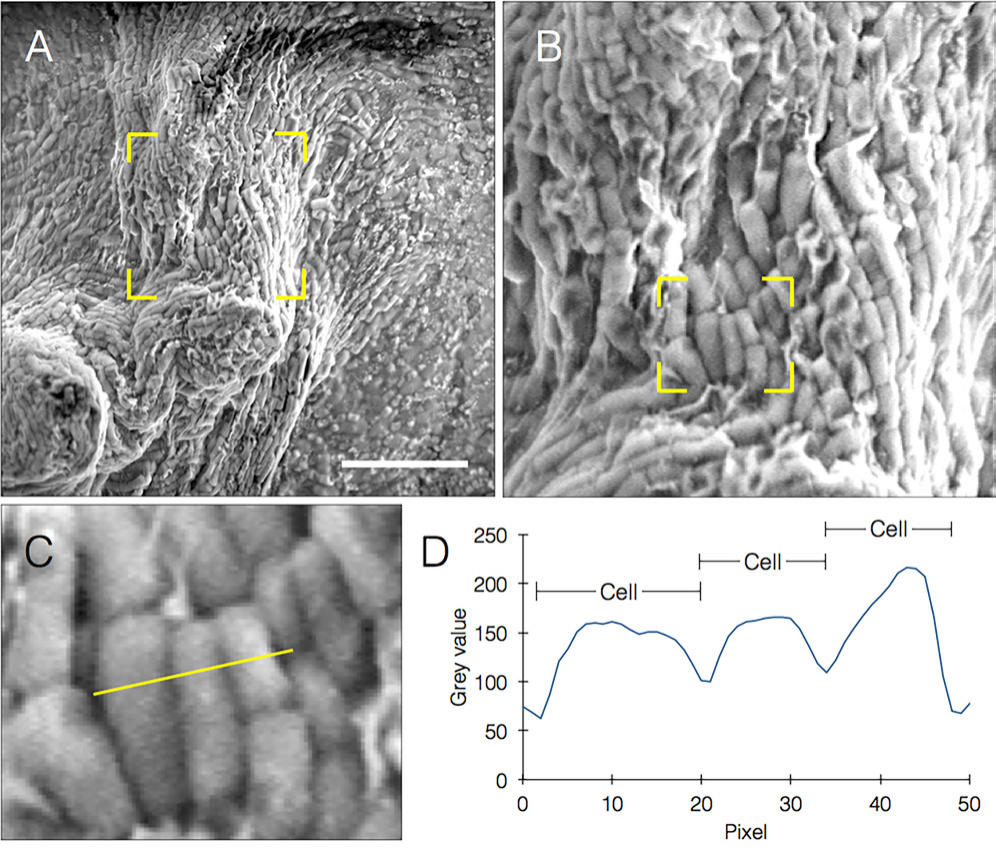
Resolving single cells in native SEM. **A.** Biofilm image acquired in native SEM. Yellow frame defines magnification area for (B) (scale bar = 10 µm). **B.** Magnified area from (A) showing closer look on single bacteria comprising this segment. Yellow frame defines magnification area for (C). **C.** Magnified single cells with yellow line drawn for profile plot. **D.** Profile plot from (C), showing three discrete peaks corresponding to bacterial cell sections.

We were able to clearly see single cells and matrix components (**Fig. 4A** and **4B**), and make accurate length measurements (**Fig. 4C** and **4D**). Almost every structural feature of the biofilm could be quantified, including the chain-like organization of bacterial cells within the biofilm and the orientations of the chain axes, length-based evaluation of the percentage of dividing cells and their locations, and areas of matrix deposition. It could also be possible to visualize both the surface and section of biofilms that have been mechanically torn (**Fig. S3**).

**Figure 5.**
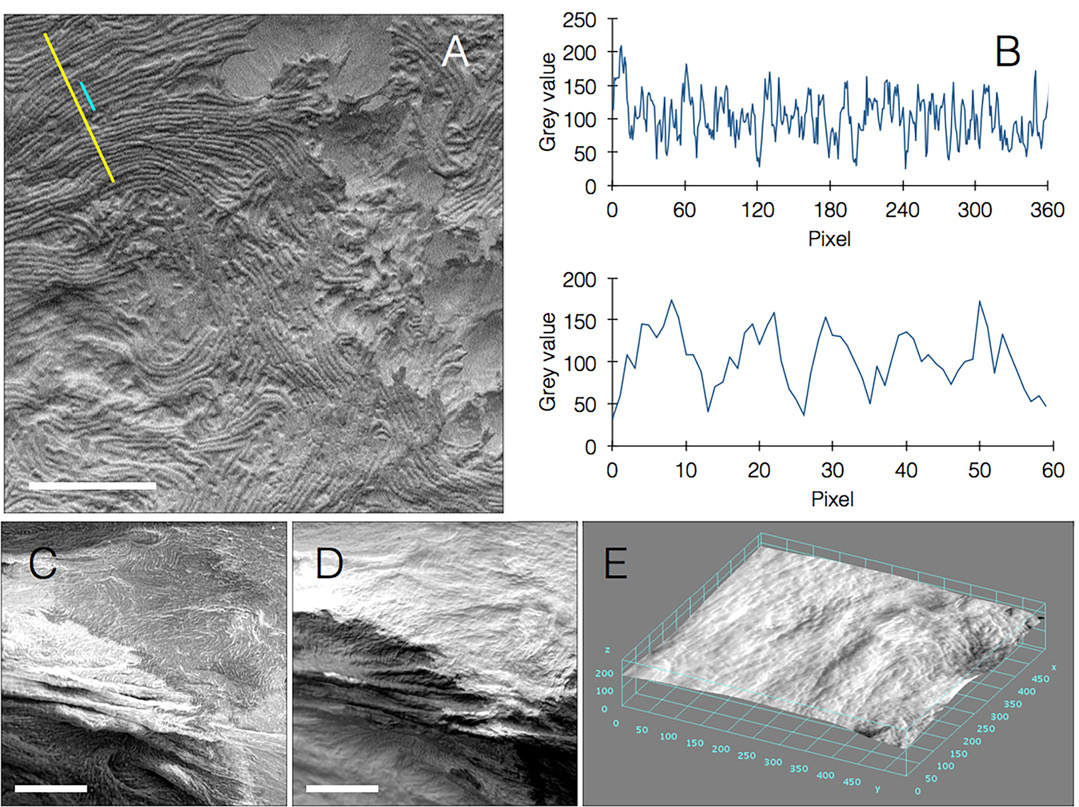
Additional modes of native SEM imaging. **A.** Imaging a frozen hydrated biofilm at −25°C, showing bacterial chains. Yellow and cyan lines define profile plots for (B) (scale bar = 30 µm). **B.** profile plots of yellow (top) and cyan (bottom) lines from (A). Peaks represent bacterial single cell-thick chains (higher details in bottom graph, representing 5 cell chains). **C-D.** Imaging at compositional and topographic modes, respectively (scale bars, **C** = 30 µm, **D** = 30 µm). **E.** 3D surface plot rendered from (D).

The ProX SEM that we used contains a variety of useful features such as an EDS module for elemental analysis, allowing to create density maps of elements across the biofilm. It has a charge-reduction sample holder, and a temperature controlled sample holder that enables imaging of samples at temperatures between 50°C and **−**25°C. We made use of this setting in rapid biofilm freezing experiments that avoid dehydration of the sample. Freezing was done rapidly on a SEM stub already held in dry ice, and in the presence of silica pellets to prevent condensation as much as possible. Although at first this caused structural artefacts (**Fig. S4**), careful calibration of this rapid, native technique resulted in high resolution images showing bacterial structures in fine detail (**Fig. 5A** and **5B** and **Fig. S5**). In addition to this, the ProX SEM enables two imaging modes: compositional (full) mode and topographic mode. Compositional mode maximizes signal in order for material information to be inferred via contrast differences, and was the main mode used in this study. In comparison, topographic mode gives an alternatively directional shading resulting in a 3D image with clearer surface structure, allowing us to create detailed 3D surface maps (**Figure 5C-5E**).

## DISCUSSION

The present study shows the result of experimenting with native biofilm imaging in SEM. Native imaging very likely preserves structural features that are inadvertently destroyed during harsher protocols of sample preparation, and its cost in time, effort, and hazard is drastically lower. It is noteworthy, that others have previously reported using rapid and pristine biofilm imaging techniques [8,9], but nevertheless the method describes here offers various advantages discussed below. **Table 1** compares native SEM imaging to other SEM techniques used for biofilm imaging.

The method is applicable to other bacterial samples as well. The process of vapor phase fixation and gentle drying is applicable to any biofilm model, with minor modifications for sample support and removal. For example, for biofilms grown inside flow cells, flow cells can be designed with removable floor on which the procedure can be applied (similar to culture flasks with microscope slide as built-in floors). For attached biofilms-glass covers/discs and any removable bacteria-compatible surface placed at the bottom and walls of the culture plate. For colonies-polycarbonate membranes placed on agar will be appropriate. With slight modifications, organs, tissues, and cell cultures could also be visualized using native SEM (**Fig. S6**).

A potential weakness of this procedure is a resolution trade-off. SEM may achieve better resolution than the one presented here [10]. For example, TasA amyloid fibers are poorly resolved in this technique. This is true in particular when using the standard protocol including fixatives and contrast agents such as osmium tetroxide; however three important points need to be noted: first, resolution is a function of instrument and not only procedure, and some instruments may produce better results than others. Second, even at somewhat lower resolutions, valuable information can be obtained from digital image analysis as shown previously [11] and as we show here. Almost every topographic feature of the B. subtilis biofilm has been reliably measured and processed. Third, required resolution is determined on the specific scientific question being studied, but it is important to weigh the benefit of preparation techniques needed to achieve optimal resolution, with their benefits. We argue here that pristine samples are inevitably more authentic, and believe that calibration of native preparation methods combined with robust image analysis and multiple acquisition modes in the instrument could very much outweigh any potential payment in resolution. Moreover, the ability to very rapidly image and analyze bacterial biofilms opens up important possibilities for high-throughput screens of biofilm-disrupting antibiotics, anti-fouling agents, *etc*.

Sample charging, a phenomenon observed in many cases using our technique, in fact turned to be an advantage in certain applications. If still undesired, it can be addressed in several routes. First, previous studies reported straightforward ways to cope with this issue [12]. In addition, the ProX could be supplemented, as mentioned above, with a charge reduction sample holder which potentially abrogates much of this effect. If this holder cannot be used, methods for viable sputtering could be devised in order to coat the upper surface of the biofilm with conducting particles. For example, immediately before drying, a colloidal suspension of conducting nanoparticles could be applied onto the biofilm surface. For certain applications the nanoparticles can be functionalized with chemical groups such as N-hydroxysuccinimide, which directly conjugates with primary amines on the bacterial surface, providing a wet form of sputtering that maintains the biofilm’s native structure.

Altogether, we report a technique for high-quality imaging of pristine, native bacterial biofilms at a fraction of the effort, time, and hazard of standard SEM methods. Native SEM could highlight features and phenotypes of bacterial biofilms, and other biological specimens, which have been nearly overlooked in the past due to lack of suitable reference imaging method. Our method is aligned with the increasing robustness of image processing and analysis algorithms, which in combination could reveal even more details than before. Improvements and adjustments of this technique could adapt it to a variety of other biological systems.

## Supplementary Material

Supplementary information **Figure S1.** Large and small scale topography of B. subtilis biofilm imaged by native SEM.
**Figure S2.** Visualizing and measuring biofilm topography in native SEM.
**Figure S3.** Visualization of both surface and section of mechanically-torn biofilm.
**Figure S4.** Sample damage caused by inappropriate freezing.
**Figure S5.** Directionality analysis performed on bacterial chains imaged at **−**25°C (**Fig. 4**).
**Figure S6.** Native SEM imaging of human liver tissue with tumor.
Supplementary information of this article can be found online at
http://www.jbmethods.org/jbm/rt/suppFiles/173.

## Figures and Tables

**Table 1.** Comparison between native SEM and other SEM methods used for biofilm imaging.

	Sample preparation	Equipment	Time	Resolution
Native SEM	Primary fixation vapor phase (GA) Drying	Desiccator	> 1 hrs	Medium to high (up to 15 nm/pixel)
Conventional SEM	Primary fixation (GA) Secondary fixation OsO4 Dehydration CPD Sputter coating	CPD device Sputter coating apparatus	hrs to days	High
Cryo SEM	Plunge frozen in slushed liquid nitrogen at −210°C Temperature raise to −95°C Temperature reduce to −125°C Sputter coating	Liquid nitrogen In vacuo transfer container Cryo preparation chamber Sputter coating apparatus	mins	Lower than conventional (good for matrix imaging)
ESEM	Non	Non	mins	Low (good for matrix imaging)
ASEM	Non	Special culture dish	mins	High Only bottom view without sample manipulation

ESEM: environmental scanning electron microscope; ASEM: atmospheric scanning electron microscope.
